# Investigating the Impact of the External Environment and Benchmark Characteristics on the China-Pakistan Economic Corridor’s Construction: A COVID-19 Perspective

**DOI:** 10.3389/fpsyg.2021.682745

**Published:** 2022-01-21

**Authors:** Aidi Xu, Abdul Hameed Pitafi, Yunfeng Shang

**Affiliations:** ^1^School of International Business, Zhejiang Yuexiu University, Shaoxing, China; ^2^School of Management, Hefei University of Technology, Hefei, China

**Keywords:** COVID-19, CPEC, CPEC external environment, CPEC development, economic stability

## Abstract

The economic stability of a country, such as Pakistan is dependent on the construction of mega-projects, such as the China-Pakistan Economic Corridor (CPEC). However, certain external factors and project characteristics may delay the construction of infrastructure projects; scholars have not investigated the development of CPEC from this perspective. In addition, the COVID-19 outbreak has hindered CPEC initiatives. This analysis will examine the effect of external environment factors on CPEC, and benchmark the project’s effects on economic stability through CPEC’s development by incorporating 523 samples obtained from employees of various CPEC projects. Structural equation modeling was used to analyze all hypotheses proposed here on AMOS 21.0 tools. According to the findings of this study, the CPEC external environment and project benchmark characteristics have a negative effect on the construction of CPEC development. Furthermore, the development of CPEC is found to have a significant effect on economic stability. However, fear of COVID-19 has weakened the relationship between CPEC development and economic stability. Finally, we also discuss the implications and limitations of the study.

## Introduction

The recent coronavirus (COVID-19) epidemic has had a disproportionate impact on underdeveloped and emerging countries, such as Pakistan, causing not only public health problems but also a debilitating economic crisis (Waris et al., 2020). According to research, the rapid dissemination of the COVID-19 virus throughout Pakistan since February 2020 has nearly halted economic development (Salik and Rafique, 2020). This is because the infection spreads rapidly and employees become sick or self-quarantine and lockdowns disturbing the market. In Pakistan, lockdown constraints have drastically reduced factory production, while quarantine, self-isolation, and work-from-home policies have reduced output, demand, and the utilization of products and services. Several researchers have studied the effects of COVID-19 on the economy (Kanwal et al., 2020a; Khan M. F. et al., 2020), health (Paakkari and Okan, 2020), education (Daniel, 2020), employment (Coates et al., 2020), and tourism (Kanwal et al., 2020c; Khan N. A. et al., 2020; Sigala, 2020); however, limited attention has been given to construction. Therefore, it is critical to understand the effect of COVID-19 on projects that are constructing the China-Pakistan Economic Corridor (CPEC). According to policymakers, CPEC promises hope and stability for Pakistan’s struggling economy (Ashraf et al., 2017; Khan et al., 2019; Kanwal et al., 2020b; Xiaolong et al., 2021). It will benefit Pakistan’s infrastructure, as well as its trade, education, and information technology sectors (Boni and Adeney, 2020): several contributions have been identified as a result of the development of CPEC. Enhanced economies have a broad influence on CPEC’s contributions (Farooqi et al., 2020; Hao et al., 2020; Nadeem et al., 2020). Developed economies significantly invest in projects to generate better returns or achieve benefits critical to boosting their economies and their people’s standard of living. Consequently, the purpose of this study is to investigate the impact of CPEC development on economic stability in the context of COVID-19.

China-Pakistan Economic Corridor consists several projects which include road and transportation construction, the development of the Gwadar port, airports, the energy sector, educational institutions, and health infrastructure (Hussain, 2018; Nand et al., 2019; Boni and Adeney, 2020). Previous research has shown that multiple projects, project density, politics, and local cultural considerations may all have an impact on a project’s development (Elawi et al., 2016; Durdyev et al., 2017; Altarawneh et al., 2018; Farooqi et al., 2021). For example, according to Durdyev et al. (2017), shortages in supply, labor, and construction materials, as well as project complexity, are the primary factors affecting infrastructure project delays. Elawi et al. (2016) found that land procurement, a shortage of expertise, and the size of projects all greatly delay development projects. Similarly, CPEC infrastructure projects face material constraints, political uncertainty, labor shortages, environmental issues, and challenges with project size (Asif and Ling, 2019; ur Rahman and ur Rehman, 2020). As a consequence of COVID-19, many companies have suspended production, workers have stayed at home, and the construction of CPEC projects has slowed. Pakistani suppliers face delays in receiving imports and in shipping exports to their destinations. Therefore, the current study’s goal is to investigate CPEC’s external environment, as well as the project benchmark characteristics of CPEC development.

COVID-19 has affected communities, enterprises, and organizations all around the world, unwittingly disrupting capital markets and the global economy. Fear of COVID-19 has resulted in high levels of anxiety, fatigue, and stress among local populations (Asempapa, 2019), with the majority of employees either affected or working from home to maintain physical distance. Due to COVID-19 fears, the Pakistani government has placed limitations on construction companies by mandating health protection policies (Pitafi et al., 2018; Sareen, 2020), thus creating demand and supply issues. At the same time, many businesses face problems of supply as governments restrict the activities of non-essential industries and employees are restricted to their residences (Pitafi et al., 2019; Jabeen et al., 2021). In the current COVID-19 scenario, when employment is at risk, many businesses have encouraged their employees to work from home; nevertheless, construction companies have on-site jobs. This all suggests that the fear of COVID-19 among employees has a negative effect on the construction industry—including CPEC projects—which negative impacts economic stability. Consequently, we propose that fear of COVID-19 acts as a boundary condition on the relation between CPEC development and economic stability.

**FIGURE 1:**
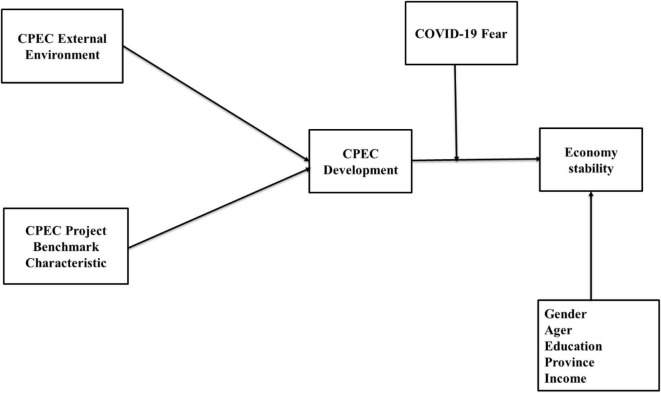
Conceptual model.

This study’s aim is to investigate the economic stability of CPEC development by using data obtained from CPEC employees. It has several theoretical and practical implications. Firstly, this study examines fear of COVID-19—a global crisis that has had a negative economic impact on the whole world. Despite this, no study has examined the effect of COVID-19 fear on construction projects, such as CPEC. Secondly, it investigates CPEC’s external environment, as well as its benchmark characteristics and their effect on CPEC development. Previous research has only concentrated on the general success of the CPEC project and its benefits to the local population (Iqbal, 2017). Thirdly, the results of this study can also guide policymakers in implementing CPEC-related policies in light of the COVID-19 outbreak. This study bridges a research gap by examining how the CPEC project is advancing economic stability. Its structured is as follows: section “Literature Review and Hypothesis Development” reviews the literature and formulates hypotheses; section “Research Methodology” contains the study’s research methodology. Section “Results and Analysis” reports the study’s findings while section “Discussion, Implications, Limitations” contains discussion, implications, and limitations. Section “Conclusion” provides a conclusion for the study. Figure 1 reflects the conceptual model of the study.

## Literature Review and Hypothesis Development

### China-Pakistan Economic Corridor External Environment and China-Pakistan Economic Corridor Development

The external environmental includes many aspects unrelated to the project but which have an effect on its success, either directly or indirectly (Ahsan and Gunawan, 2010; Zawawi et al., 2011; Wibowo and Alfen, 2014). Scholars have identified a number of environmental concerns—political, legal, social, and financial—that can affect a project’s development (Jin et al., 2012; Pitafi et al., 2020a). Windapo and Cattell (2013) proposed that external environmental factors, such as weather and climate could have an effect on development over all levels of a project’s life cycle. Research has also found that external environmental factors can trigger time delays or even a project’s termination during the construction stage (Yong and Mustaffa, 2013; Zou et al., 2014). Theoretical literature indicates that the external environment has a negative impact on project construction.

As with other construction projects, external environmental factors influence CPEC construction. Its route passes through all of Pakistan’s provinces, including Gilgit-Baltistan (Abid and Ashfaq, 2015). According to officials, CPEC is not the name of a single road project but consists of multiple projects (Ashraf et al., 2017). In addition, the political environment in Pakistan has been volatile over the last decade, with political parties casting doubt on CPEC’s construction (Ali, 2016; Pitafi et al., 2020b). Furthermore, the Baloch tribes are opposed to the construction of the CPEC scheme, believing that it is only a route to occupy their rich territory (Nabi et al., 2018; Ul Hassan, 2020). Chinese workers have, on occasion, been kidnapped and killed in Baluchistan while Baloch separatists have attacked tankers transporting fuel to a Chinese mining company. Furthermore, the United States (USA), India, and Israel are all opposed to the construction of CPEC (Ali, 2015). India believes that, with the construction of CPEC, Pakistan will become economically stronger and could present the Kashmir problem before the United Nations (UN). Based on previous studies, this research suggests the following hypothesis:

**H1a:** CPEC’s external environment has a negative impact on CPEC development.

### China-Pakistan Economic Corridor Project Benchmark Characteristics and China-Pakistan Economic Corridor Development

The most important factors of construction projects are their length, importance, operations, density, and priority (Zou et al., 2014; Altarawneh et al., 2018). It should also be noted that construction projects, which can comprise several activities, may operate beyond their contractual deadline (Li et al., 2012; Altarawneh et al., 2018). On the other hand, several studies have discovered that project leader performance can affect development projects (Altarawneh et al., 2018; Rashid et al., 2020). Research has suggested that if a project has tasks that are common rather than complicated (Gudienė et al., 2013), the project leader will schedule and track project activities. Furthermore, the size and availability of resources affect a project’s development. For example, equipment, skilled workers, their availability, and project leadership often have effects over time, resulting in budget overruns and project delays.

China-Pakistan Economic Corridor is a mega-project that consists of several construction projects. Its construction is delayed for a variety of reasons, such as equipment and materials shortages, conflicting routes, and a lack of qualified labor (Kanwal et al., 2020c). For example, CPEC transportation projects have two routes: eastern and western (Sun et al., 2020). It is believed that the western route has a higher priority, government allocations are more focused on it rather than the eastern route (Aslam et al., 2016; Sun et al., 2020). In addition, CPEC’s route passes through much agricultural land: local populations are concerned that international investors could take it over (Ashraf et al., 2017; Kanwal et al., 2019b; Wei et al., 2020). The route also passes through Gilgit Baltistan, where the construction of the CPEC route is adversely affected by weather—especially during winter. Similarly, the route passes through the desert regions of Baluchistan and Sindh provinces, where the summer weather causes more delays. Furthermore, the role of bureaucracy is critical in construction projects; in Pakistan, bureaucratic interference in CPEC’s construction process is causing delays in development (Wolf, 2020; Kurita, 2021). This study therefore proposes the following hypothesis:

**H1b:** CPEC’s project benchmark characteristics have a negative impact on CPEC development.

### China-Pakistan Economic Corridor Development and Economic Stability

Pakistan presently faces energy crises, which severely affect its economy. Its weakened economy has produced high inflation, resulting in widespread poverty (Allauddin et al., 2020). In order to resolve Pakistan’s energy shortage, the government has prioritized the energy sector in CPEC programs (Kanwal et al., 2020a). It is expected that CPEC projects will play a major role in the country’s economic development (Khalique et al., 2020). Both Chinese and Pakistani authorities regard CPEC as a game-changing project, with 27 economic zones spread throughout both countries that will support industrialization, economic growth, and several developments in the energy sector in Pakistan (Raza et al., 2018; Boni and Adeney, 2020). Scholars and policymakers have also stated that CPEC not only promotes trade between China and Pakistan but also with the rest of the world (Kanwal et al., 2020b; Saad et al., 2020). With the development of mega-infrastructure and regional connectivity, the host community will benefit economically from jobs, business, and easy access to the global market (Kanwal et al., 2019a). According to officials, CPEC construction can attract international investors and bring direct foreign investment to Pakistan through various manufacturing sectors, resulting not only in local infrastructure development but also economic development (Abid and Ashfaq, 2015; Wang, 2017). Ali (2018) concluded that CPEC will boost industrial development and connect Pakistan’s business industries internationally, benefiting the domestic economy. Specifically, CPEC road and transport construction will connect rural areas of Pakistan with major towns, thus increasing the connectivity of local populations in villages with cities, facilitating them economically.

Research has indicated that China’s investment in CPEC is a potential game-changer for Pakistan’s economy (Kamran et al., 2020; ur Rahman and ur Rehman, 2020). Although blessed with natural resources, Pakistan’s power and energy industries are underperforming. CPEC can configure and improve its economy’s comparatively poorly performing industries, enhancing Pakistan’s power in several respects (Saoud, 2019). CPEC is an evolving driver that can promote the development of several industrial zones; these will have a major multi-dimensional effect on the economy of local populations (Ali et al., 2018). CPEC can help stabilize the energy demands of Pakistani industries through different energy projects which can also boost the local economy (Moin and Qadri, 2020). Research has also found that CPEC is a game-changing initiative for Pakistan, with the ability to boost its low economic situation and generate thousands of new job opportunities for its population (Wolf, 2018; Khan and Liu, 2019). On the basis such research, this study proposes the following hypothesis:

**H2:** CPEC development has a positive impact on economic stability.

### Moderating Role of Fear of COVID-19

The recent COVID-19 outbreak has afflicted the whole world, including Pakistan, and has been responsible for more than 100,000 deaths globally (Freudendal-Pedersen and Kesselring, 2020). COVID-19 has harmful effects for the daily lives of individuals and can have serious and even fatal results (Ahorsu et al., 2020). However, the complete extent of COVID-19’s impact is still unknown; it has already created significant societal instability, economic disruption, and public health problems since it spread across the globe. COVID-19 was discovered in Wuhan, China at the end of 2019. Since then, people’s daily routines have changed, partly due to fear of COVID-19 due to its rapid rate of contamination and relatively high mortality. Some people have underlying health problems which make them more susceptible to respiratory infections like COVID-19, such as asthma and immunosuppression (Raghubir and Latimer, 2014). Such people may need to take a high level of precautions to keep “safe,” including staying at home and maintaining physical distance. This study assumes that COVID-19 fear may plays a moderating role in the relationship between CPEC development and economic stability.

Research indicates that the consequences of the COVID-19 pandemic are not restricted to health but can have a significant influence on economies like Pakistan’s (Mamun and Ullah, 2020; Nicola et al., 2020), with CPEC construction projects facing suspension. Fear of COVID-19 is creating economic difficulties for those whose lives and families have been affected as a result of lockdowns in Pakistan (Islam et al., 2021c). Furthermore, low income countries, such as Pakistan could be facing more serious crises than do developed countries. COVID-19 related constraints, including physical distancing, self-isolation, and work from home (Gkiotsalitis and Cats, 2021), have a greater impacting on CPEC construction (Nadeem et al., 2020). Among other problems, Chinese engineers and labor have endured travel bans from other countries due to the COVID-19 epidemic (Yan and Berliner, 2011; Christidis and Christodoulou, 2020; Wen et al., 2020; Younis et al., 2020). In addition, COVID-19 may also induce psychological mediators, including sadness, fear, anger, annoyance, frustration, helplessness, and anxiety among individuals. The key explanations for its negative impacts on CEPEC’s development are restrictions on travel and the movement of products across the region, as well as COVID-19 fear. Fear associated with COVID-19 can, therefore, be identified as a moderator construct on the link between CPEC development and economic stability such that, when COVID-19 fear is higher, the significant link between CPEC development and economic stability is weaker. This study therefore proposes the following hypothesis:

**H3:** COVID-19 fear negatively moderates the relationship between CPEC development and economic stability such that higher the COVID-19 fear, the lower the relationship between CPEC development and economic stability.

## Research Methodology

### Data Collection Procedures

To better understand the aim of the current research, the authors adopted a quantitative approach that gathered data from the employees of various CPEC projects throughout Pakistan. These employees were the primary respondents of this study; they were contacted via email. Due to travel restrictions and local lockdowns in Pakistan, we collected data for this study online using Google’s survey platform. All the respondents were notified that their data would only be used for research purposes and that it would be kept confidential. The questionnaire is split into three parts. The first was a cover letter that explained the study’s purpose and certain measurement item concepts that were included in the research model. The demographic details of participants were requested in the second section. The final portion includes the measurement items for all of the constructs. The author also informed the participants that the survey is voluntary and that they could exit it at any time. We developed the questionnaire in English since it is an official language of Pakistan and is easily understood by its educated population. In the context of CPEC, the wording of certain constructs has been slightly modified. The author firstly invited a faculty member from the university’s English department to review the survey’s English accuracy, readability, and any grammatical errors. Secondly, the author invited several faculty members from the management and economic departments to assist with the survey material’s corrections and recommendations. After their suggestions, some survey items were modified. The author used a convenient sampling approach because it was easy to contact educated respondents using this method. In addition, the author requested the participants to complete the questionnaire and circulate its link among fellow CPEC employees. We initially performed a pilot study with 43 responses, and its findings were considered to be reliable, with Cronbach’s alpha (CA) being greater than 0.700.

From June 2020 to September 2020, we circulated 750 questionnaires among employees of CPEC projects. CPEC’s higher management assisted us by forwarding the questionnaire to other employees. We received 620 responses over 4 months, with a response rate of 82.66%. Some of these responses were not included in the final dataset because they were completed incorrectly or some entries were left blank. Consequently, the final dataset comprised 523 responses. The profile of participants surveyed is shown in Table 1. According to this table, respondents are over the age of 20 and educated. This analysis was performed on educated employees since they have sufficient knowledge about CPEC projects. Furthermore, less-educated employees have difficulty filling out surveys and are unaware of the long-term advantages of CPEC. Table 1 includes detailed information about all the respondents.

**TABLE 1:** Demographic information of the samples.

Variables	N	Percentage	Variables	N	Percentage
**Gender**			**Location**		
Male	327	62.50	Punjab	118	22.60
Female	196	37.50	Sindh	100	19.10
**Age**			KPK	89	17.00
21–30 years old	184	35.20	Baluchistan	91	17.40
31–40 years old	132	25.20	Gilgit Baltistan	77	14.70
41–50 years old	106	20.30	Islamabad	48	9.20
>50 years old	101	19.30	**Income**		
**Education Level**			10,000–25,000 rupees	161	30.80
College degree or below	184	35.20	26,000–50,000 rupees	165	31.50
Bachelor’s degree	215	41.10	51,000–75,000 rupees	125	23.90
Master’s degree or higher	124	23.70	76,000–100,000 rupees	42	8.00
			>100,000 rupees	30	5.00

### Research Instruments

Several metrics were used to achieve the research objectives. All measurements and scales were taken from previous studies that were well-known and well-structured in their respective domains (Latif et al., 2021). The author used a five-point Likert scale that ranged from “strongly agree” to “strongly disagree” (Wu et al., 2021). researchers have indicated no variation in outcomes from using a five- or seven-point scale (Pitafi et al., 2020a), so we used a five-point scale. Five constructs were used in the research model: economic stability, COVID-19 fear, CPEC development, CPEC external environment factors, and CPEC project benchmark characteristic. The constructs are detailed below.

#### Economic Stability

In this study, the dependent variable was economic stability. This was calculated using total of six items with a scale taken from Ali et al. (2018) and Kanwal et al. (2020a). The example item is “CPEC holds great promise for my community’s future.”

#### COVID-19 Fear

This construct was used as a moderator variable in this study, with a scale used from Ahorsu et al. (2020). COVID-19 fear consists of six items; the sample item for this scale is “I worry a lot about COVID-19.”

#### China-Pakistan Economic Corridor Development

This construct was used as a mediator variable in this study and consists of five items with a scale borrowed from Kanwal et al. (2020c) and Saad et al. (2019). The sample item for this scale is “CPEC will develop the quality of roads.”

#### China-Pakistan Economic Corridor External Environment

This was used as an independent construct in the research model. The items for CPEC external environment consisted of five items and were adapted from Altarawneh et al. (2018). The sample item is “There are complexity and uniqueness in CPEC project activities.”

#### China-Pakistan Economic Corridor Project Benchmark Characteristic

This was used as an independent variable in the research model and consisted of five indicators adapted from Altarawneh et al. (2018). The sample item is “There are physical environment problems in CPEC projects like location and works.”

#### Control Variables

To measure the actual impact of independent variables on the dependent variable age, gender, education, location, and income were used as a control variable.

## Results and Analysis

### Assessment of Bias

The author investigated the common method bias (CMB) problem for the survey data using the guidelines from previous research. Researchers recommend several procedures to solve possible CMB problems (Pavlou and El Sawy, 2006; Podsakoff et al., 2012; Wu et al., 2021). The author initially adopted several technical remedies, including the anonymity of the replies as previous researchers have suggested that response privacy could help resolve CMB. Next, the author used several statistical methods to resolve the probability of CMB in the dataset. Following the procedure of Liang et al. (2007), the outcome revealed that the substantive factor had a score of 66.4% of the variance, while the method factor had a score of 1.2% of the variance, indicating no possibility of CMB in the current data. Furthermore, Table 2’s output revealed that all constructs had inter co-relationships less than 0.600 (Pavlou and El Sawy, 2006), indicating that the study did not suffer from CMB. Finally, the author conducted a variance inflation factor (VIF) analysis, with the results indicating that the VIF value is smaller than the recommended score of 3.33 (Latif et al., 2019; Pitafi et al., 2020b), again suggesting that CMB did not occur in the data set. All the evidence presented above indicated that CMB did not exist in this study.

**TABLE 2:** Means, standard deviation, and correlations.

Variable	M	SD	1	2	3	4	5	6	7	8	9	10
1 CPEC project external environment	2.283	0.698	**0.723**									
2. CPEC project benchmark characteristic	2.579	0.981	0.026	**0.718**								
3. CPEC development	3.500	0.824	–0.108	–0.401	**0.762**							
4. Economy stability	3.799	0.858	–0.200	–0.240	0.274	**0.722**						
5. COVID-19 fear	2.370	0.864	0.075	0.479	–0.392	–0.174	**0.747**					
6. Income	**NA**	**NA**	–0.007	0.113	–0.118	–0.002	0.124	**NA**				
7. Location	**NA**	**NA**	–0.028	0.022	0.029	–0.193	–0.137	–0.469	**NA**			
8. Education	**NA**	**NA**	0.105	0.082	0.069	–0.045	0.051	0.125	–0.111	**NA**		
9. Age	**NA**	**NA**	–0.171	–0.128	0.034	0.122	–0.180	–0.406	0.244	–0.502	**NA**	
10. Gender	**NA**	**NA**	0.120	–0.183	0.139	–0.072	–0.154	–0.285	0.240	–0.200	0.230	**NA**

*M, Mean; SD, Standard division.*

*Diagonal elements are AVE square root.*

### Validity and Reliability

To statistically analyze the conceptual model of this study, we used AMOS version 21.0 and SPSS version 21.0. Following the procedures mentioned in previous literature (Fornell and Larcker, 1981; Hair et al., 2019; Pitafi et al., 2020a), we first computed the reliability of the conceptual model using Cronbach’s alpha (CA), composite reliability (CR), and the average variance extracted (AVE). Scholars believe that CA is appropriate when it is greater than 0.700. Table 3 revealed that all variables had CA scores ranging from (0.763–0.844) > 0.700. CR is applicable if the value is greater than 0.700; Table 3 shows that all constructs have CR scores ranging from (0.836–0.883) > 0.700. Finally, AVE is appropriate when it has values greater than 0.500; Table 3 shows that AVE varies from (0.516–0.581). Furthermore, the convergent validity of the study model was found by utilizing factor loading (Nadeem et al., 2021). Scholars indicate that the loading could be greater than 0.600 for satisfactory convergent validity of the suggested model (Wu et al., 2020). Table 3 shows that all items for all variables have a loading higher than the minimum suggested value of 0.600 (Latif et al., 2020), ranging from (0.610–0.878). Therefore, the proposed research model of the study has an acceptable degree of reliability and convergent validity.

**TABLE 3:** Results of confirmatory factor analysis.

Construct	Items	Loading	CA	CR	AVE	MSV	ASV
CPEC project external environment	5	0.610–0.754	0.788	0.845	0.523	0.040	0.014
CPEC project benchmark characteristic	5	0.675–0.824	0.763	0.836	0.516	0.229	0.112
CPEC development	5	0.646–0.878	0.798	0.845	0.581	0.161	0.100
Economy stability	6	0.654–0.795	0.816	0.864	0.522	0.075	0.051
COVID-19 fear	6	0.610–0.818	0.844	0.883	0.559	0.229	0.105

*CA, Cronbach’s alpha; CR, composite reliability; AVE, average variance extracted; MSV, Maximum shared variance, ASV, Average shared variance.*

*Discriminant validity: ASE < MSV.*

The discriminant validity of the research model was assessed using the results of Tables 2, 3. Table 3 shows that all variables have ASV values less than MSV, indicating an acceptable degree of discriminant validity (Pitafi and Ren, 2021). Table 2 reveals that the inter-co-relation values of all of the constructs are less than the AVE square root, indicating that the conceptual model has acceptable discriminant validity (Fornell and Larcker, 1981).

### Hypotheses Testing

The proposed model of the current study was analyzed using AMOS 21.0 software (Hair et al., 2017; Islam et al., 2021c) and a structural equation technique of maximum likelihood was used. The results of structural equation modeling confirmed that all model fit values (CFI = 0.916, TLI = 0.902, IFI = 0.917, NFI = 0.868, AGFI = 0.889, REMSA = 0.053, CMIN/DF = 689.31/279 = 2.47) are within the recommended range (Hair et al., 2017). In addition, Table 4 depicts the results of structural equation modeling, which all indicate that the present data supports all the proposed hypotheses. The findings show that the CPEC external environment (*B* = –0.115, *p* < 0.05) and CPEC project benchmark characteristic (*B* = –0.335, *p* < 0.001) have negative CPEC development, validating H1a, and H1b. CPEC development has a positive effect on economic stability (*B* = 0.710, *p* < 0.001), validating H2. H1a, H1b, and H2 are thus confirmed by the existing data; therefore, both findings suggest that beta scores are in the suggested range, and that t values are greater than 1.64 and p values for all relationships are less than 0.05. Furthermore, it was discovered that all the control variables had an insignificant impact on economic stability.

**TABLE 4:** Hypothesis1 testing.

Path	Standard coefficient	*t*-value	Result
CPEC project external environment to CPEC development	–0.115	–3.47**	Significant
CPEC project benchmark characteristic to CPEC development	–0.355	4.80**	–
CPEC development to economy stability	0.710	–5.88**	–
COVID-19 fear to economy stability	–0.756	–6.86***	–
CPEC development* COVID-19 fear	–0.257	–4.78**	Significant
Income to economy stability	0.02	0.94	Insignificant
Location level to economy stability	0.01	1.79	–
Education to economy stability	0.46	1.05	–
Age to economy stability	0.54	1.08	–
Gender to economy stability	0.71	1.81	–

*p < 0.05, **p < 0.01, ***p < 0.001.

### Moderation Analysis

We also hypothesized that COVID-19 fear will have a negative impact on the relationship between CPEC development and economic stability. The relationship between CPEC development and economic stability would be weakened as COVID-19 fear levels rise. Table 4 reveals that COVID-19 fear has a negative interaction with CPEC development, and economic stability (*B* = –0.257, *p* < 0.001), supporting H3.

**FIGURE 2:**
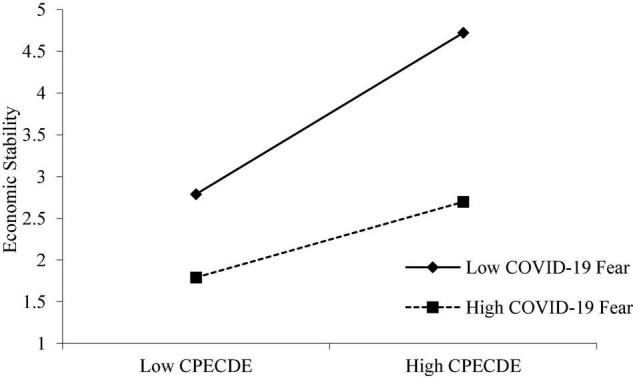
Moderating effect of COVID-19 Fear in the relationship between CPEC development and economic stability. CPECDE, CPEC development.

Furthermore, we used a graphical technique (Aiken et al., 1991) to examine the moderating function of COVID-19 fear. Figure 2 shows that COVID-19 fear weakens the connection between CPEC development and economic stability. Figure 2 depicted two stages of COVID-19 fear: higher and lower levels of it. As expected, the relationship between CPEC development and economic stability weakens as COVID-19 fear increases. In other words, COVID-19 fear reduces the relationship between CPEC development and economic stability.

## Discussion, Implications, Limitations

### Discussion

The current study’s aim is to investigate the impact of CPEC development on economic stability in the context of COVID-19, utilizing data obtained from CPEC project employees. According to this research, the impacts of the COVID-19 pandemic are not restricted to human health but also have a major impact on construction projects and the economy (Paakkari and Okan, 2020; Rasheed et al., 2020; Sareen, 2020; Trougakos et al., 2020). The current data validated all the suggested hypotheses. According to our assumptions, the results show that the CPEC external environment and CPEC project benchmark characteristics have a negative effect on CPEC development, validating H1a and H1b. As CPEC is a mega-project, it consists of many schemes that are currently under development in Pakistan (Ashraf et al., 2017; Ali, 2020; Islam et al., 2021a). Each province of Pakistan has a unique geographical location, culture, and political environment. Baloch tribes oppose the development of CPEC projects because they believe it is only a way to occupy their land. Furthermore, Baloch tribes have their own lifestyle and traditions. For example, Kanwal et al. (2020c) stated that the majority of the Gwadar area’s residents work in the fishing industry; however, with the development of the new Gwadar port, they are no longer permitted to fish in the deep sea. Similarly, the political parties of Pakistan are in conflict about the construction of the CPEC route. Previous studies have also made similar findings in the context of different construction projects (Jin et al., 2012; Islam et al., 2021b). In addition, previous research has revealed that environmental, political, geographic, social, financial, and natural factors (Jin et al., 2012; Cao et al., 2020) have an impact on construction projects. Also linked to a project’s development are its type, significance, operations, density, and priority (Yong et al., 2014; Zou et al., 2014; Altarawneh et al., 2018). This study’s findings have also confirmed the proposed H2, that CPEC development has a significant impact on economic stability. Prior studies have also reported economic benefits from the construction of CPEC to Pakistan and its community (Raza et al., 2018; Ali et al., 2020).

Furthermore, this study’s results suggest that COVID-19 fear weakens the relationship between CPEC development and economic stability—H3—which is also confirmed by the current dataset. Previous research has also shown that fear of COVID-19 is causing economic hardship for those individuals whose lives and families have been disrupted as a consequence of lockdown in Pakistan (Sun et al., 2020; Islam et al., 2021c). Lockdowns limit the supply of products from factories, and quarantine and self-isolation have a negative effect on the development of CPEC projects.

### Implications

This research has several implications for theory and practice. Firstly, it investigates the development of CPEC construction and its economic impact through the CPEC external environment and its project benchmark characteristics. It supplements previous research, which has examined the impact of CPEC development or the overall the benefits of CPEC projects (Khalique et al., 2020; Saad et al., 2020; Lai et al., 2021) but ignored the external environment and the CPEC project characteristics that are directly related to the construction of CPEC projects. Secondly, this study’s results indicate that CPEC development has a significant impact on economic stability. It is well-known that the construction industry has a positive impact on the economy of a country as well as on local populations. Therefore, CPEC officials must be focused on how CPEC construction promotes economic stability.

Thirdly, the current study emphasizes the importance of COVID-19 fear as a moderating role in the relationship between CPEC development and economic stability. Individuals are experiencing psychological mediators, such as depression, distress, anger, frustration, disappointment, isolation, and anxiety in relation to COVID-19. This study’s results indicate that COIVID-19 weakens the relationship between CPEC development and economic stability. CPEC officials must implement world standards of COVID-19 protection which are also important for CPEC project construction.

Finally, the author recommends that CPEC officials consider external environment factors and CPEC benchmark characteristics for the smooth construction of their projects. The federal government of Pakistan could engage all regional stockholders in the development of CPEC projects and distribute them equally across all provinces. It could also negotiate with Baloch tribes to address their suspicions. Social media can also play an important role in the development of CPEC; officials should post and share the progress of construction projects on social media to ensure the proper and successful development of CPEC.

### Limitations

While the current study has several implications, it still has several drawbacks that should be addressed in future research. Firstly, this analysis does not investigate the mediating role of CPEC development. In addition, we examined COVID-19 fear as a moderator in this study. Future research should include a mediating construct, such as CPEC project management, as well as other moderating variables, and analyze its effect on economic stability. Secondly, the author collected data from CPEC project employees across Pakistan. With thousands of Chinese workers employed in various CPEC projects, the delay and suspension of CPEC’s construction due to COVID-19 has had an economic effect on them: future research should thus include Chinese workers, which could yield more interesting outcomes.

One downside of this research is its limited sample type: the majority of participants in this study are educated employees. However, many workers or illiterate staff are employed on daily wages in various CPEC projects and they are also economically affected by COVID-19 and Pakistan’s lockdown situation. Future research could conduct face-to-face interviews with such employees.

Finally, we used a survey methodology to gather data for this study. Its results may therefore be explained in the light of the shortcomings of such a research tool, generally recognized as survey error (Eckman and Leeuw, 2017). Hence, future research should consider testing the research model by utilizing other design techniques, including tests and observations, to improve the reliability and validity of the results (Guinea and Markus, 2009).

## Conclusion

This study has investigated the impact of the construction of CPEC on economic stability by collecting data from employees working in different CPEC projects. CPEC is a mega-project which not only economically benefits both countries but also local communities. Moreover, the construction of CPEC is also affected by the outbreak of COVID-19, which has serious affected the economy. This study’s results have validated all the suggested hypotheses. Specifically, its outcome indicated that CPEC’s external environment and CPEC project benchmark characteristics have a negative impact on the construction of CPEC development. CPEC development has a significant impact on economic stability; however, fear of COVID-19 weakens the relationship between CPEC development and economic stability.

## Data Availability Statement

The raw data supporting the conclusions of this article will be made available by the authors, without undue reservation.

## Ethics Statement

The studies involving human participants were reviewed and approved by Hefei University of Technology China. The patients/participants provided their written informed consent to participate in this study.

## Author Contributions

AX, AP, and YS conducted this research in collaboration. AX designed the overall research model. AP collected the data, analyzed the data, and wrote the methodology section. YS reviewed the final version and helped in the literature writing process. All authors contributed to the article and approved the submitted version.

## Conflict of Interest

The authors declare that the research was conducted in the absence of any commercial or financial relationships that could be construed as a potential conflict of interest.

## Publisher’s Note

All claims expressed in this article are solely those of the authors and do not necessarily represent those of their affiliated organizations, or those of the publisher, the editors and the reviewers. Any product that may be evaluated in this article, or claim that may be made by its manufacturer, is not guaranteed or endorsed by the publisher.
